# Assessing the impact of pain-linked Nav1.7 variants: An example of two variants with no biophysical effect

**DOI:** 10.1080/19336950.2020.1870087

**Published:** 2021-01-25

**Authors:** Kim Le Cann, Jannis E. Meents, Vishal Sudha Bhagavath Eswaran, Maike F. Dohrn, Raya Bott, Andrea Maier, Martin Bialer, Petra Hautvast, Andelain Erickson, Roman Rolke, Markus Rothermel, Jannis Körner, Ingo Kurth, Angelika Lampert

**Affiliations:** a Institute of Physiology, RWTH Aachen University Hospital, Aachen, Germany; b Department of Neurology, Medical Faculty, RWTH Aachen University Hospital, Aachen, Germany; c Division of Clinical Metabolism of Medical Genetics and Human Genomics at Northwell Health System, New-York, United States; d Department for Palliative Care, Medical Faculty, RWTH Aachen University, Aachen, Germany; e Department of Chemosensation, AG Neuromodulation, Institute for Biology II, RWTH Aachen University, Aachen, 52074, Germany; f Department of Anaesthesiology, Medical Faculty, RWTH Aachen University, Aachen, Germany; g Institute of Human Genetics, Medical Faculty, RWTH Aachen University Hospital, Aachen, Germany

**Keywords:** Erythromelalgia, pain, orthostatic hypotension, nav1.7 mutation, hek293t cells, patch-clamp recordings, inherited pain, sodium channel, homology modeling

## Abstract

Mutations in the voltage-gated sodium channel Nav1.7 are linked to human pain. The Nav1.7/N1245S variant was described before in several patients suffering from primary erythromelalgia and/or olfactory hypersensitivity. We have identified this variant in a pain patient and a patient suffering from severe and life-threatening orthostatic hypotension. In addition, we report a female patient suffering from muscle pain and carrying the Nav1.7/E1139K variant. We tested both Nav1.7 variants by whole-cell voltage-clamp recordings in HEK293 cells, revealing a slightly enhanced current density for the N1245S variant when co-expressed with the β1 subunit. This effect was counteracted by an enhanced slow inactivation. Both variants showed similar voltage dependence of activation and steady-state fast inactivation, as well as kinetics of fast inactivation, deactivation, and use-dependency compared to WT Nav1.7. Finally, homology modeling revealed that the N1245S substitution results in different intramolecular interaction partners. Taken together, these experiments do not point to a clear pathogenic effect of either the N1245S or E1139K variant and suggest they may not be solely responsible for the patients’ pain symptoms. As discussed previously for other variants, investigations in heterologous expression systems may not sufficiently mimic the pathophysiological situation in pain patients, and single nucleotide variants in other genes or modulatory proteins are necessary for these specific variants to show their effect. Our findings stress that biophysical investigations of ion channel mutations need to be evaluated with care and should preferably be supplemented with studies investigating the mutations in their context, ideally in human sensory neurons.

## Introduction

The voltage-gated sodium channel Nav1.7, encoded by the *SCN9A* gene, is preferentially expressed in sensory neurons of dorsal root ganglia (DRGs) and sympathetic ganglia [1]. Its key role in human pain signaling is impressively demonstrated by loss-of-function Nav1.7 mutations causing congenital insensitivity to pain [2], while gain-of-function mutations lead to severe painful disorders, such as inherited erythromelalgia (IEM) [3], paroxysmal extreme pain disorder [4], or small fiber neuropathy (SFN) [5]. Fibromyalgia is characterized by multi-focal pain comprising allodynia and hyperalgesia. It may be accompanied by myalgia, lower back pain, or headache [6]. SFN is a neuropathic pain disorder in which small diameter nerve fibers are affected while large diameter fibers are spared [5]. IEM is an autosomal dominant neuropathy characterized by severe burning pain and redness in the distal extremities, which leads to severe impairment of the patient’s everyday life.

Sodium channels are responsible for the fast upstroke of the action potential and are thus in an ideal position to regulate excitability of the expressing cells. There are nine different Nav α subunits described to date, which can be associated with β subunits that modulate their cell membrane expression and their gating [7,8].

Here, we characterize two Nav1.7 variants identified in pain patients to better understand their potential role in the pathophysiology of the patients’ phenotypes. We identified the heterozygous Nav1.7/N1245S variant in one patient diagnosed with SFN and in one patient with severe and treatment-resistant orthostatic hypotension as well as sexual and sudomotor dysfunction. Two further patients, carrying the same heterozygous variant have been described in the literature: a 50-year-old female patient with burning pain in both feet, a low threshold of pain detection, hyperalgesia and increased olfactory sensitivity [9] and a 67-year-old male patient with burning pain in the feet and connective tissue disease [10]. In a further study, six patients out of a cohort of 393 patients were diagnosed with SFN and carried the N1245S variant [11]. The variant (chr2:167,094,638 GRCh37) was classified as a variant “with unknown significance.” It has also been suggested to be a single nucleotide polymorphism [12].

We also describe a novel heterozygous variant, Nav1.7/E1139K (chr2:167,108,299 GRCh37), in a female patient of 39 years who suffers from severe myalgia exacerbated by exercise. She also had been diagnosed with fibromyalgia. The variant is rare and only reported in 7 out of 248,222 alleles (Genome Aggregation Database).

Reported Nav1.7-IEM mutations often shift the channels’ activation toward more hyperpolarized potentials, thus supporting enhanced excitability of the expressing dorsal root ganglia neurons [13]. This may lead to the pain symptoms reported by the patients. Our electrophysiological results suggest that both Nav1.7 variants do not interfere with gating properties nor with the structural conformation in a way that would directly support their role in the pain pathophysiology. These data point to the limitations of the heterologous expression method and advocate a careful evaluation of effects observed in such studies.

## Materials and methods

### Patients

The patient carrying the p.E1139K variant was examined in the Division of Medical Genetics at Northwell Health System, USA (Table 1, patient 6), and two patients carrying the p.N1245S variant were examined at the Neuromuscular Outpatient Clinic and/or the Outpatient Clinic of Disorders of the Autonomic Nervous System, Department of Neurology of the RWTH Aachen University Hospital, Aachen, Germany (Table 1, patients 1 and 2). The respective diagnosis was confirmed by a detailed patient history, neuropathic pain questionnaires, a neurological examination, and sensory and motor nerve conduction studies of the lower limbs to exclude a large fiber polyneuropathy. Extensive laboratory tests were performed to rule out any acquired cause of neuropathy. Patient 1 underwent quantitative sensory testing and a skin biopsy obtained at the distal lower limb, whereas patient 2 was examined by autonomic testing with head-up tilt and cardiovagal tests.

**Table 1.** Patients/subjects with the Nav1.7/N1245S or the Nav1.7/E1139K variant. Characteristics of the variant carriers, pain phenotype, olfactory sensitivity, and comorbidities are described

Patient No	Nav1.7 Variant	Sex	Age of onset	Pain/Diagnosis	Olfactory sensitivity	Concomitant diseases	References
**1**	N1245S	Male	20	Small fiber neuropathy	not assessed	Recurrent diarrhea	Dr Maike Dohrn, Aachen
**2**	N1245S	Male	62	No	not assessed	Severe autonomic failure with orthostatic hypotension, dry skin, dry eyes, erectile dysfunction, coat hanger pain	Dr Andrea Maier, Aachen
**3**	N1245S	Female	40	IEM	Hyperosmia	No	[9]
**4**	N1245S	Male	60	Burning pain	not assessed	Connective tissue disease	[10]
**5**	N1245S	Unknown 6 subjects from two families	Unknown	No	not assessed	No	[11,12]
**6**	E1139K	Female	39	Myalgia, pain in the extremities with reddening, jaw pain	not assessed	Hypotonia, visual problems, intestinal paralysis	Dr. Martin Bialer, USA

### Genetic studies

The study is in accordance with the Helsinki Declaration and was approved by the local ethics board of the RWTH University Hospital Aachen (research ethics approval EK302–16). We obtained the patients’ written consent prior to study inclusion. HaloPlex libraries for NGS were prepared using the HaloPlex Target Enrichment kit for custom design (1–500kb target region, Agilent Technologies) according to the manufacturer’s protocols. Target sequences (exons) and adjacent intronic (±50 bp) sequences were enriched. The gene panel included known disease-causative genes for hereditary pain disorders (genes implicated in HSAN1-HSAN8, and CIP) as well as functional candidate genes (genes encoding TRP-channels, ASICs, neurotrophins, and their receptors). Libraries were run on a MiSeq Sequencer (Illumina) with a 300 cycle MiSeq reagent kit v2 (Illumina). Details are available upon request.

### Homology modeling and structural analysis of variants

The cryo-EM structure of the hNav1.7-β1-β2 complex (PDB ID 6J8G) was used as a template for homology modeling [14]. The template structure was prepared for homology modeling by removing all the heteroatoms and the β2 subunit. The N1245S variant sequence was generated and aligned to the hNav1.7 using Jalview [15]. One hundred models were generated using MODELLER 9.22 [16]. The DOPE-HR scores obtained from MODELLER and visual inspection with the template were used to choose the best model. Analysis of the intramolecular interaction partners for amino acids of interest in hNav1.7 and N1245S variant were studied using PyMol with a distance cutoff of 4 Å. The E1139K variant could not be modeled, since the intracellular linker between DII and DIII has not been resolved in the template structure.

### Plasmid, DNA cloning, and mutagenesis

For expression in mammalian cells, hNav1.7/N1245S and hNav1.7/E1139K variants were generated based on WT human Nav1.7 (*SCN9A* transcript variant 1 containing 1277 amino acids, NM_002977.3) in a modified pCMV6-neo vector by site-directed mutagenesis using Q5 Polymerase (NEB). Constructs were verified by commercial DNA sequencing (Eurofins, Germany). The GFP-tagged full-length human Navβ1 subunit was expressed in a pCLH vector.

### Expression of hNav1.7 and hβ1 in mammalian cells

The human embryonal kidney HEK293T cell line was used as a heterologous expression system. It was cultured in Dulbecco’s Modified Eagle’s Medium (DMEM, Gibco Life Technologies, Germany), supplemented with 10% fetal bovine serum (Biochrom AG, Germany) and 1% penicillin/streptomycin (Life Technologies, Germany). HEK293T cells were seeded 2 to 24 hours before transfection. Transfection was performed with JetPei (PolyPlus-transfection SA, Peqlab, Germany) using 1.25 µg *SCN9A* plasmid DNA and 0.25 µg pMaxGFP plasmid. For some experiments with the Nav1.7/N1245S variant, a GFP-tagged construct of the β1 subunit was co-transfected. In that case, 0.25 µg β1 DNA were added. Cells were recorded 24 hours after transfection. Only fluorescent cells were used for recordings.

### Patch-clamp experiments

HEK293T cells were recorded at room temperature (22 ± 2°C) using an EPC 10 USB patch-clamp amplifier (HEKA Electronics, Germany). Sampling rates were set to 50 kHz (100 kHz for deactivation protocols) and a 10 kHz low-pass filter was used (30 kHz for deactivation protocols).

Recording patch pipettes were manufactured with a DMZ puller (Zeitz Instrument GmbH, Germany) and had a tip resistance between 1.2 and 2.5 MΩ. The external bath solution contained the following (in mM): 140 NaCl, 3 KCl, 1 MgCl_2_, 1 CaCl_2_, 10 HEPES, 20 glucose. The pH was adjusted to 7.3 using NaOH, and osmolarity was 300–310 mOsm. The patch pipette internal solution contained (in mM): 10 NaCl, 140 CsF, 1 EGTA, 10 HEPES, 18 sucrose; pH was adjusted to 7.3 and osmolarity to 300–310 mOsm.

Series resistance was compensated by at least 70%. Only recordings with a series resistance below 6 MΩ were analyzed. Leak current was subtracted online with the P/4 procedure. For all protocols, the holding potential (*V*_hold_) was set at −120 mV unless otherwise stated. A liquid junction potential of +8.3 mV was corrected in all experiments. After establishing the whole-cell configuration, inward Na^+^ currents were allowed to stabilize for 5 min during repeated 0 mV depolarization steps before starting the recordings. Current density was obtained by dividing the maximum peak current (pA) by the cell capacitance (pF).

The voltage dependence of activation was assessed from holding potential using 40 ms pulses (test-pulse) to a range of test potentials from −100 mV to +50 mV in 5 mV or 10 mV incremental steps with an interval of 5 s. For conductance–voltage curves, the reversal potential (*V*_rev_) for each individual neuron was determined. Conductance (*G*) was calculated at each voltage (*V*) using the equation *G*= *I*/(*V–V*_rev_) with *V*_rev_ being the reversal potential for sodium and *I* being the inward current at the respective voltage (*V*). Conductance–voltage curves were fitted using a Boltzmann equation: *G* = *G*_max_/(1 + exp [(*V*_m –_ *V*_1/2_)/*k*]), where *G*_max_ is the maximum sodium conductance, *V*_m_ is the membrane voltage, *V*_1/2_ is the potential of half maximum channel activation and *k* is the slope factor. The voltage error during the current-voltage relationship was calculated for each recording. The mean voltage error was not significantly different between groups.

The voltage dependence of steady-state fast inactivation was measured using a two-step protocol. A 500 ms pre-pulse with various potentials ranging from *V*_hold_ to +10 mV in 5 or 10 mV incremental steps was used to inactivate the channels. This pre-pulse was immediately followed by a 40 ms test-pulse at 0 mV to determine the remaining fraction of available channels. Inward current measured during the test-pulse to 0 mV was normalized to the cell’s maximum test-pulse inward current. The normalized peak inward current amplitude at each test-pulse is displayed as a function of pre-pulse potential and fitted using the above Boltzmann equation.

The time course of fast inactivation estimates how quickly channels undergo fast inactivation. The regular current-voltage (I–V) protocol described above was used to activate and inactivate channels. Current decay was fitted by a double-exponential equation: Y(t) = (Y_0_ – Y_tx_)*exp(-K*X) + Y_tx_, where Y_0_ is the current amplitude at time 0, Y_tx_ is the Y value at infinite times, K represents the rate constant and X is the time. The time constants τ_fast_ and τ_slow_ are the reciprocals of the respective rate constant K. They are plotted against the test-pulse voltage.

Recovery from fast inactivation was investigated using a two-pulse voltage protocol with a recovery inter-pulse of varying duration in between. Cells were first depolarized to +10 mV for 500 ms from *V*_hold_. This depolarizing pre-pulse (*I*_pre_) was immediately followed by the inter-pulse to −120 mV. The duration of this step increased with each sweep by a factor of 2, from 0.1 ms to 1.638 s. The inter-pulse was followed by a depolarizing test-pulse (*I*_test_) at +10 mV for 40 ms to assess the number of recovered channels. The current amplitude at the test-pulse *I_test_* was normalized to the current amplitude measured at the pre-pulse *I_pre_* and plotted against inter-pulse duration and fit with a double exponential equation: Y = Y_0_+ A_fast_*(1_exp[K_fast_*X])+A_slow_*(1_exp[K_slow_*X]), where Y_0_ is the current amplitude at time 0, A_fast_ and A_slow_ represent the amplitude coefficient for the fast and the slow time constants, K_fast_ and K_slow_ represent the two rate constants and X is the time. The time constants τ_fast_ and τ_slow_ are the reciprocals of the respective rate constant K.

The voltage dependence of steady-state slow inactivation was investigated using a four-pulse voltage protocol. Cells were first depolarized by a reference pulse (*I*_ref_) to +10 mV for 10 ms from a holding potential of −120 mV (N1245S) or −140 mV (E1139K). This reference pulse allows a comparison of current amplitudes over long periods, taking time-dependent changes into account. The reference pulse was followed by a 500 ms recovery step and, subsequently, by a range of 30 s pre-pulses at varying voltages from −140 mV to +40 mV using a 20 mV increment. A −120 mV (N1245S variant) or −140 mV (E1139K variant) inter-pulse of 200 ms allowed the channels to recover from fast inactivation before the standard 40 ms test-pulse at +10 mV (*I*_test_). For current analysis, peak test-pulse current amplitudes were normalized to their respective reference-pulse current (*I*_test_/*I*_ref_) and plotted against pre-pulse voltage, providing a sigmoidal curve that was fitted with the above Boltzmann equation.

Onset of slow inactivation was assessed using a three-step voltage protocol. From a holding potential of −140 mV, the cell membrane was depolarized to +10 mV for 10 ms. This reference pulse (*I*_ref_) was followed by a 500 ms step at −140 mV before an inter-pulse depolarized the cell membrane again at +10 mV during 0.1 s to 73 s in six sweeps. Finally, a 40 ms test-pulse (*I*_test_) at +10 mV followed a 200 ms repolarization step at −140 mV and measured the current amplitude of the available channels. For the current analysis, peak test-pulse current amplitudes were normalized to their respective reference-pulse current (*I*_test_/*I*_ref_) and plotted against inter-pulse duration.

The time course of channel deactivation was investigated using a two-pulse voltage protocol. A pre-pulse of +20 mV for 0.4 ms allowed maximum current development while avoiding fast inactivation. This brief activation pre-pulse was followed by a 100 ms repolarization from *V*_hold_ up to +10 mV in 10 mV increments to induce tail currents. After each sweep, the channels were allowed to recover for 5 s. The time constant of deactivation was determined by a single exponential fit to the decay of tail currents.

Use-dependency was determined by the application of 30 depolarizing pulses to 0 mV at a frequency of either 1, 10, 30, 50, or 100 Hz. Sodium peak inward current amplitude was measured at each pulse and normalized to the current amplitude of the first pulse to determine the use-dependent decline of inward current amplitude. For quantification, the area under the curve (AUC) for each graph was calculated and compared.

### Data analysis and statistics

Data were analyzed and graphed using Fitmaster software (HEKA Elektronik), Igor Pro software (Wavemetrics), GraphPad Prism 5 or 6 (GraphPad Software), SPSS (IBM SPSS Statistics Version 25), and Corel Draw X6 (Corel Corporation).

For statistical testing, two groups were compared with a Student’s *t* test for parametric testing or a Mann–Whitney U test, depending on normal distribution. More than two groups were compared by an ANOVA, followed by a Bonferroni’s multiple comparison post hoc analysis. The exact value of n (number of cells) is indicated in the figure legends. Data are presented as mean and error bars denote 95% confidence interval (CI). No outliers were defined or eliminated.

## Results

### Clinical description of patients

Two subjects were treated at the Department of Neurology of the University Hospital RWTH Aachen, Germany: patient 1 is a 41-year-old male, who displayed burning and stabbing pain as well as hyperesthesia in legs and feet at the age of 20 years (Table 1). Clinical examinations, quantitative sensory testing, and a distal skin biopsy confirmed the diagnosis of a small fiber neuropathy. Nerve conduction studies of the lower limbs did not show any measurable large fiber involvement. Extensive laboratory tests were negative for any known acquired cause. The family history was positive as the patient’s father and his 15-year-old daughter reported similar symptoms, suggesting an autosomal dominant mode of inheritance.

The second male patient (patient 2, Table 1) was 62 years old at the time of examination, presenting with severe and treatment-resistant orthostatic hypotension, erectile dysfunction, dry eyes and skin, coat hanger pain, cold feet and mostly red, warm hands, hypohidrosis, but no neuropathic pain phenotype. His symptoms began when he was about 58 years old. Autonomic testing revealed severe and combined cardiovagal and sympathoneural failure with only 5 min standing time. Both patients’ exomes were sequenced and identified as carrying a heterozygous c.3734A>G variant (NM_002977) in exon 20 of the *SCN9A* gene, leading to the Nav1.7/N1245S variant. This variant was associated with pain symptoms [9,10], but it also occurs in individuals without any pain phenotype [11].

For the N1245S variant in *SCN9A*, the preponderance of available in-silico prediction programs (BayesDel noAF, DANN, DEOGEN2, EIGEN, EIGEN PC, FATHMM, FATHMM-XF, LIST-S2, LRT, MVP, MetaLR, MetaSVM, MutationTaster, PROVEAN, REVEL, and SIFT) provided a pathogenic/deleterious verdict, while it was considered tolerated by four additional tools (BayesDel addAF, Mutation assessor, PrimateAI, and SIFT4G). As in-silico prediction tools are specifically prone to loss-of-function, these predictions might not fully cover the gain-of-function pathomechanism expected to be underlying in these gain-of pain phenotypes, however. With an allele frequency of 1.224/278.244 (0.44%) and 5 homozygous carriers (Genome Aggregation Database), a gene–disease relationship can only be argued when assuming a reduced penetrance and/or variable expressivity. In the ClinVar database, the variant is therefore classified by “conflicting interpretations of pathogenicity.”

A 39-year-old female patient (patient 6, Table 1) suffering from severe myalgia exacerbated by exercise reported to the Division of Medical Genetics at Northwell Health System, New York, United States. She describes intense pain in the extremities along with reddening, as well as jaw pain. She has been diagnosed with fibromyalgia and displays also other symptoms, such as hypotonia from childhood, visual impairments, and intestinal paralysis requiring subtotal colectomy and 3-methylglutaconic and 3-methylglutaric aciduria. She has been examined using whole-exome sequencing and she was found to carry the heterozygous c.3415 G > A variant (NM_002977) in the *SCN9A* gene, leading to the Nav1.7/E1139K variant.

### The N1245S substitution leads to alternative intramolecular interaction partners

**Figure 1.**
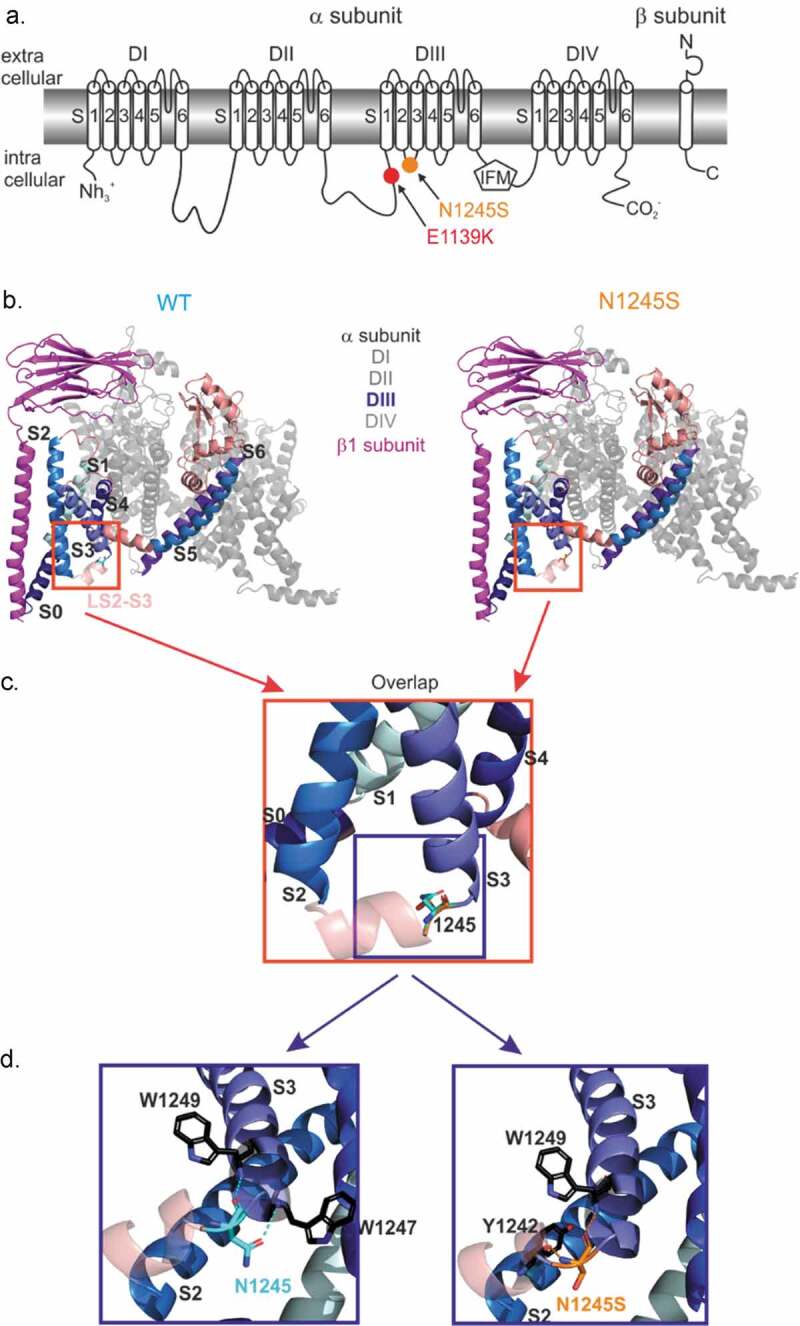
**Nav1.7/N1245S and Nav1.7/E1139K variants are both located in intracellular loops of the Nav1.7 protein**. (a) The N1245S variant is found in the intracellular linker between DIII S2 and S3 and E1139K is located in the large intracellular linker between DII and DIII. (b) Visualization of the hNav1.7 WT (left, light blue) and the N1245S variant (right, orange) protein structure associated with the β1 subunit (magenta) using PyMol. DI, DII and DIV are depicted in gray and DIII-DIV linker in salmon. DIII S0 to S6 segments are labeled in a range of blue colors. The S2-S3 linker (LS2-S3) of DIII is indicated in light salmon. (c) A magnified overlap view of (B) is shown with the variant N1245S in orange and the WT N1245 in light blue. DIII segments are labeled in black. (d) A detailed, slightly anti-clockwise turned view of the overlap in (C) reveals non-covalent bonds between N1245 (WT, left) or N1245S (variant, right) and two possible interaction partners. DIII segments are labeled in black

The two variants Nav1.7/N1245S and Nav1.7/E1139K are localized in intracellular loops of the Nav1.7 channel (Figure 1a). To investigate if structural changes within the sodium channel protein are likely to be induced by the N1245S variant, we studied the molecular structural conformation of both Nav1.7 WT and variant protein, located in the intracellular loop of domain III between segments 2 and 3 Figure 1(b-d). The E1139K variant is located in the intracellular linker between domains II and III, which was not resolved in the CryoEM structure [14].

The interaction partners of the template structure (WT channel, Figure 1b-d left) and homology model (N1245S variant, Figure 1b-d right) were investigated using PyMol. The intramolecular interaction partners of the sidechains of asparagine N1245 (WT channel, Figure 1b-d left) and the serine S1245 (variant channel, Figure 1b-d right) were investigated. Both amino acids are polar uncharged hydrophilic amino acids, but the serine is smaller in size than the asparagine. We focused on interaction partners located at a distance of 0 to 4 Å to the 1245 locus. Generally, it should be noted that distances of 2.2–2.55 Å are thought to be “strong, mostly covalent,” while distances of 2.5–3.22 Å are “moderate, mostly electrostatic” and distances of 3.2–4.0 Å are defined as “weak, electrostatic” [17,18]. We identified two interaction partners for each isoform of the channel (Figure 1d). Nav1.7 WT and its variant shared one interaction partner, a tryptophan (W1249), which resides at the beginning of DIII S3 and is 3.55 Å from the 1245 locus. We also identified one interaction partner specific to the WT N1245: a tryptophan W1247, 3.22 Å from the variant (Figure 1d left) and one interaction partner specific to the variant S1245: a tyrosine Y1242, 3.55 Å from the variant (Figure 1d right). Both are located in the intracellular loop between DIII S2 and S3. All these interactions can be considered as weak electrostatic interactions; therefore, it is not clear how this change in molecular interactions would affect channel function. Nevertheless, the variant’s position at the intracellular linker between S2 and S3 may allow for interaction with intracellular proteins or other modifications. It should be noted, however, that the substitution of an asparagine by a serine in the Nav1.7/N1245S variant did not lead to the creation of a novel phosphorylation consensus site (supplementary data).

### The Nav1.7/N1245S variant co-expressed with β1 increases current density

**Figure 2.**
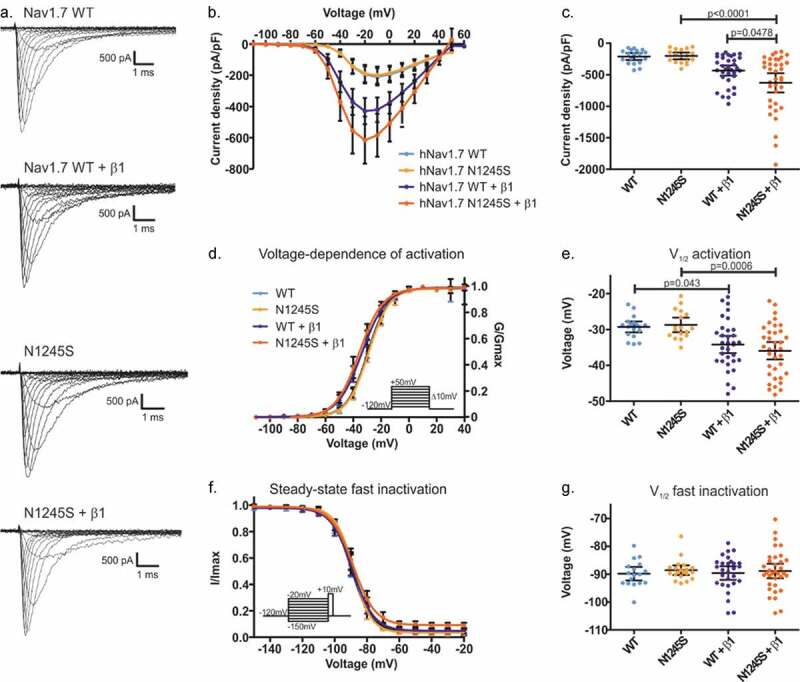
Current density and gating properties of the hNav1.7/N1245S variant co-expressed with the β1 subunit. (a) Representative traces of sodium current of Nav1.7 WT, N1245S variant, WT + β1 subunit and N1245S + β1 subunit. The scale bar depicts 500 pA and 1 ms. (b) Current density-voltage relationship for the four conditions (Nav1.7 WT in light blue, n = 18, N1245S variant in light orange, n = 17, Nav1.7 WT + β1 in dark blue, n = 33, N1245S + β1 in dark orange, n = 36). (c) Values of current density for the four conditions. (d) Conductance-voltage relationship. Voltage dependence of activation protocol is indicated in the inset. (e) V_*1/2*_ values of activation, obtained based on data in (d). (f) Voltage dependence of steady-state fast inactivation. The protocol is indicated in the inset. (g) The displayed V_*1/2*_ values were obtained based on the data in (f). See Table 2 for the exact values. All data are shown as mean ± 95% confidence interval

To decipher its potential pathological mechanisms, we investigated the gating properties of Nav1.7/N1245S as compared to the WT channel when transiently expressed in HEK cells. When expressed alone, no effect by the N1245S variant on current density, voltage dependence of activation, or steady-state fast inactivation was observed (Figure 2).

The β1 subunit is expressed in small-diameter primary sensory neurons [19]. The recent Cryo-EM structure of Nav1.7 together with the β1 subunit showed that the transmembrane domain of β1 resides close to the voltage-sensor of DIII, the area of the channel which contains the N1245 [14]. We therefore co-expressed the β1 subunit along with Nav1.7/N1245S or WT and looked for a potential change in current density or voltage-dependent gating.

We found a statistically significant difference in the current density between groups (F(3, 101) = 9.35, p < 0.0001) (Figure 2c). The mean peak sodium current density in Nav1.7 WT was increased when Nav1.7 was co-expressed with the β1 subunit (mean diff. = 426 pA/pF, 95% CI 187.2 to 665.5 pA/pF, p < 0.0001) as shown by Bonferroni’s multiple comparison test. An increased mean peak sodium current density of Nav1.7 in the presence of the β1 subunit was also observed for the N1245S variant (mean diff. = 195.5 pA/pF, 95% CI 1.141 to 389.8 pA/pF, p = 0.0478) (Figure 2b, c and Table 2). We also found a significant difference in the voltage dependence of activation by the N1245S variant compared to WT when co-expressed with the β1 subunit (F(3, 100) = 5.91, p < 0.0001) Figure 2(d, e). When co-expressed with the β1 subunit, we detected a potentiating effect in both WT and variant channels, shifting the potential of half-maximal activation (V_1/2_) to more hyperpolarized potentials (mean diff. = 4.88 mV, 95% CI 0.09 to 9.67 mV, p = 0.043 between WT and WT+β1, and mean diff. = 7.22 mV, 95% CI 2.4 to 12.0 mV, p = 0.0006 between N1245S and N1245S+β1). This potentiation by the β1 subunit affected both WT and N1245S in a similar way (Figure 2d, e and Table 2). We also investigated steady-state fast inactivation of Nav1.7, but found insufficient evidence to support an effect by the N1245S variant or by co-expression of the β1 subunit (F(3, 95) = 2.679, p = 0.051) (figure 2f and g and Table 2).

**Table 2.** Current density as well as voltage dependence of activation and steady-state fast inactivation of Nav1.7 WT or N1245S variant with or without β1 co-expression. Values are given as mean and upper and lower 95% confidence intervals are presented in brackets

	**WT**	**N1245S**	**WT +** β**1**	**N1245S +** β**1**
**Cell number**	18	17	34	36
**Current amplitude (pA)**	−3574 (−4663 to −2485)	−3853 (−5048 to −2658)	−4589 (−5534 to −3644)	−5928 (−7321 to −4535)
**Current density (pA/pF)**	−209.4 (−264.7 to −154.1)	−201 (−255.7 to −146.3)	−431.9 (−513.7 to −350.1)	−627.3 (−778.7 to −476.0)
**Activation** **- V_1/2_ (mV)** **- Slope**	−29.3 (−30.8 to −27.8) 7.4 (6.8 to 7.9)	−28.7 (−30.8 to −26.7) 7.3 (6.7 to 7.9)	−34.2 (−36.6 to −31.8) 7.9 (7.4 to 8.3)	−35.9 (−38.4 to −33.5) 7.5 (7.2 to 7.9)
**Inactivation** **- V_1/2_ (mV)** **- Slope**	−89.8 (−92.3 to −87.4) 5.9 (5.5 to 6.4)	−88.6 (−90.3 to −86.8) 5.4 (5.0 to 5.8)	−89.6 (−92.0 to −87.2) 5.8 (5.4 to 6.1)	−88.9 (−91.4 to −86.3) 5.4 (5.0 to 5.7)

Finally, no persistent current was recorded in the relevant voltage range in either Nav1.7 WT or its variant form and the time-to-peak of the sodium inward current was similar for both channel isoforms (Fig. S1).

### Neither N1245S variant nor β1 affects use-dependent current decline

Rodent nociceptors can fire up to 70 Hz within a burst [20], so we decided to investigate whether the N1245S variant affects the use-dependent decline of current amplitude in Nav1.7 and could thereby have an effect on nociceptor firing frequency and pain signaling. We decided to simulate different firing rates: 1 Hz, 10 Hz, 30 Hz, 50 Hz, and 100 Hz.

**Figure 3.**
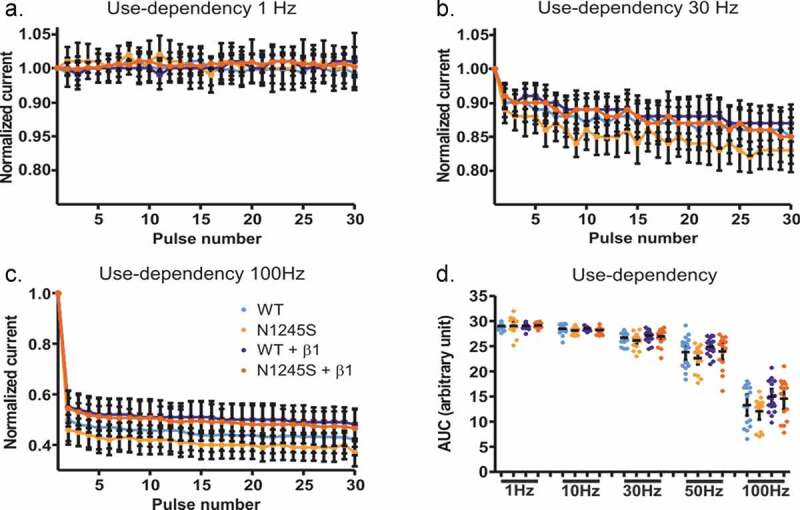
The use dependency is similar for the WT and the variant channel N1254S.(a-c) HEK293T cells expressing either Nav1.7 WT, Nav1.7 N1245S, Nav1.7 WT + β1 or Nav1.7 N1245S + β1 were stimulated with 30 depolarizing pulses to 0 mV at a frequency of 1 Hz (a), 30 Hz (b), or 100 Hz (c). (d) The area under the curve (AUC) was calculated for each condition for statistical comparison between the different groups. WT in light blue, n = 18; N1245S in light orange, n = 17; WT + β1 in dark blue, n = 18; N1245S + β1 in dark orange, n = 16. Inward current measured at each pulse was normalized to the current amplitude from the first pulse. Error bars denote 95% confidence interval. SeeTable 3 for exact values

At a depolarization frequency of 1 Hz, no use-dependent current decline could be observed in any group (Figure 3a). At 10 Hz, we observed a 2% use-dependent current run-down at the 30^th^ pulse as compared to the current amplitude at the first pulse (Fig. S2). The current decline was more pronounced at higher frequencies, with 40% to 60% at 100 Hz Figure 3(b, c). However, no significant difference was detected between WT and N1245S either with or without β1 (F(19, 316) = 8.11, p > 0.99) (Figure 3d, Table 3).

**Table 3.** **Area under the curve (AUC) of Nav1.7 in the presence or absence of β1 at 1, 10, 30, 50, or 100 Hz**. Values are given as mean, upper and lower 95% confidence interval indicated in brackets

	Nav1.7 WT	Nav1.7/N1245S	WT + β1	N1245S + β1
1 Hz	29.0 (28.7 to 29.3)	29.0 (28.2 to 29.8)	29.1 (28.8 to 29.3)	29.1 (28.9 to 29.4)
10 Hz	28.5 (28.0 to 29.0)	28.1 (27.7 to 28.5)	28.5 (28.4 to 28.7)	28.2 (27.9 to 28.5)
30 Hz	26.7 (26.1 to 27.3)	26.2 (25.4 to 26.9)	27.1 (26.5 to 27.7)	27.0 (26.1 to 27.8)
50 Hz	23.8 (22.3 to 25.4)	22.7 (21.4 to 24.0)	24.9 (24.1 to 25.7)	24.0 (22.2 to 25.7)
100 Hz	13.3 (11.1 to 15.4)	12.1 (10.4 to 13.7)	15.0 (13.5 to 16.5)	14.6 (12.5 to 16.7)

### The N1245S variant does not affect gating kinetics

Some Nav1.7-IEM mutations have been reported to alter fast inactivation and/or deactivation kinetics [21]. As both processes may alter neuronal excitability, we decided to investigate both of these processes in the Nav1.7/N1245S variant.

**Figure 4.**
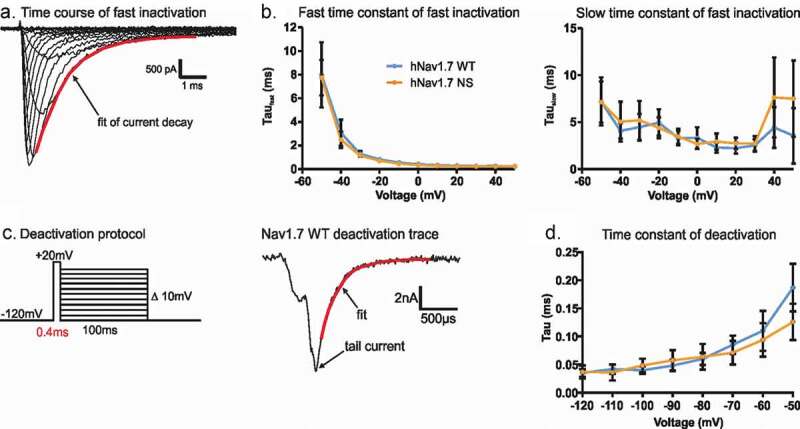
The N1245S variant does not affect Nav1.7 kinetics of fast inactivation and deactivation. (a) Double exponential fit (red) to the current decay was used to investigate the time constants of channel inactivation. (b) τ_fast_ (left) and τ_slow_ (right) obtained by double exponential fit of fast inactivation as shown in (a). WT in blue, n = 18; N1245S in orange, n = 17. (c) Deactivation protocol (left). Example trace (right) of a tail current at −60 mV obtained during the repolarization step from the deactivation protocol. The red line represents the single exponential fit. (d) Time constant of deactivation was obtained by single exponential fit of the decay of the tail currents. Error bars denote 95% confidence interval. These recordings were performed without co-transfection of the Nav β1 subunit

To measure the time course of channel inactivation, we used a double exponential fit to the inward current decay during a depolarizing stimulus (Figure 4a). Both channel isoforms displayed virtually identical time constants. Neither the fast time constant (τ_fast_, e.g. at −20 mV: 0.72 ± 0.0002 ms and 0.82 ± 0.0002 ms for the variant and the WT channels, respectively), nor the slow time constant (τ_slow_, e.g. at −20 mV: 4.4 ms for the variant and 4.99 ms for the WT channels) were affected by the variant (Figure 4b). This indicates that both channel isoforms undergo fast inactivation with a similar time course.

The closing of the activation gate was investigated by determining the time constant of deactivation with a single exponential fit. Nav channel activation was induced by a very short depolarizing pre-pulse to activate as many channels as possible while minimizing the percentage of channels undergoing fast inactivation (Figure 4c, left). This brief activating pre-pulse was then followed by a hyperpolarizing pulse to induce tail currents at varying potentials (Figure 4c, right). The single exponential fit to the decay of tail currents again revealed similar kinetics of deactivation for both channel isoforms, e.g. 0.094 ± 0.014 ms for the variant and 0.11 ± 0.017 ms of the WT channels at −60 mV (Figure 4d).

### The N1245S variant enhances slow inactivation

**Figure 5.**
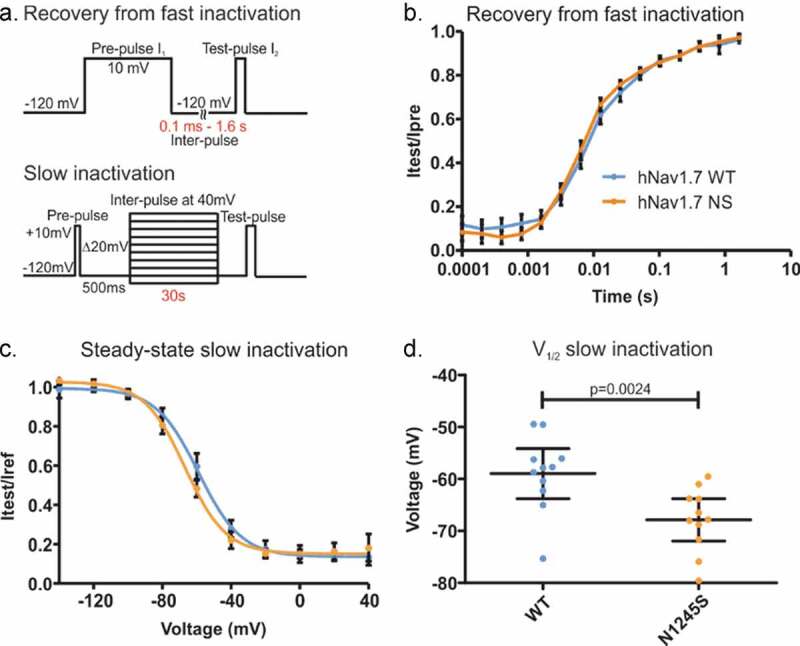
The N1245S variant enhances slow inactivation. (a) Voltage protocols of recovery from fast inactivation (top) and slow inactivation (bottom). (b) Time course of recovery from fast inactivation. Peak current at the test-pulse (I_2_) was normalized to the peak obtained during the pre-pulse (I_1_) and plotted against inter-pulse duration using a logarithmic scale. WT in blue, n = 32; N1245S in orange, n = 23. (c) Voltage dependence of steady-state slow inactivation. Peak current at the test-pulse was normalized to the peak obtained during the pre-pulse and plotted against the inter-pulse voltage and fit with a Boltzmann function. WT in blue, n = 11; N1245S in orange, n = 11. (d) V_1/2_ values of slow inactivation were obtained based on the data in (c). Error bars denote 95% confidence interval. These recordings were performed without co-transfection of the Nav β1 subunit

Quicker recovery from fast inactivation and enhanced slow inactivation are gating properties common to many Nav1.7-IEM mutations [22–25]. We used a two-pulse protocol with increasing inter-pulse duration to investigate recovery from fast inactivation (Figure 5a). However, we found no difference between channel isoforms, e.g. at 6.4 ms of inter-pulse at −120 mV, 45.8 ± 2% of recovery for the variant, and 41.5 ± 2% recovery for the WT channels (Figure 5b). This means that the N1245S variant does not affect the recovery process.

Next, we used 30 s depolarizing stimuli to drive channels into slow inactivation across various voltages (Figure 5c) and measured the half-maximal slow inactivation potential (V_1/2_). Slow inactivation was enhanced in the variant Nav1.7/N1245S channel. We found a −9.95 ± 2.8 mV hyperpolarized shift in the V_1/2_ of slow inactivation of the N1245S variant compared to the WT channel (U = 16, p = 0.0024) (Figure 5d). This means that variant channels inactivate at potentials closer to the resting membrane potential, which would constitute a loss-of-function.

### The new Nav1.7/E1139K variant does not affect voltage-dependent gating or current density

We investigated another potential chronic pain variant of Nav1.7:E1139K, identified in a patient suffering from severe myalgia exacerbated by exercise. Although the exact mechanisms of fibromyalgia remain unclear, it is known that the development of this chronic pain condition results from augmented sensory and pain processing [6]. To decipher whether the Nav1.7/E1139K variant may be responsible for the chronic pain state of the female patient, we performed whole-cell voltage-clamp recordings in HEK cells expressing either Nav1.7 WT or its E1139K variant. The variant is located in the large intracellular linker between DII and III (Figure 1). This large amino acid loop has not been resolved in the cryo-EM structure of the channel [14], so it is difficult to predict its role in channel function. Since the intracellular linker is not known to interact with β subunits, we decided to investigate the channel without co-expression of these subunits.

**Figure 6.**
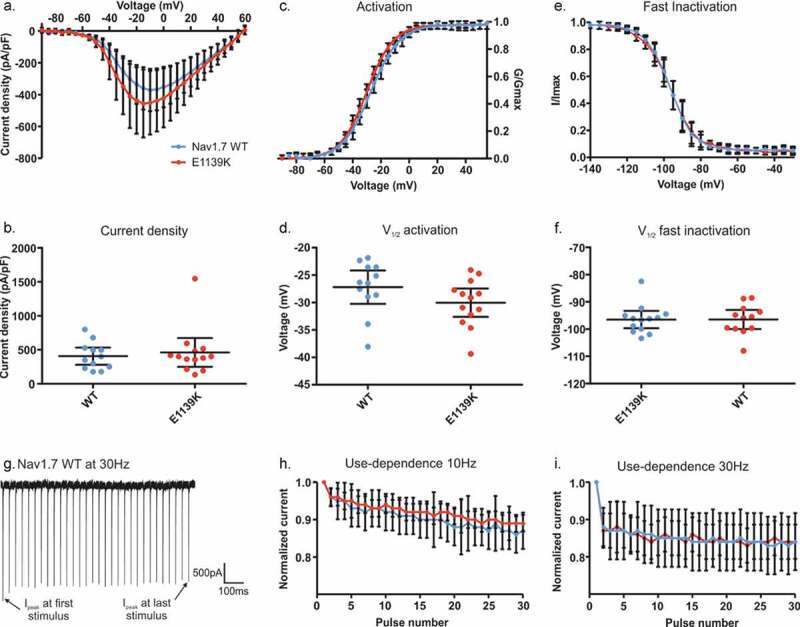
**E1139K and Nav1.7 WT channels display no differences in activation, fast inactivation and use-dependency**. (a) Current-voltage relationship for E1139K variant (red, n = 13) and Nav1.7 WT (blue, n = 12) channels. (b) Values of current density for the two conditions. (c) Conductance-voltage relationship. Conductance of each cell was normalized to the same cell’s peak conductance to fit the data with a Boltzmann function. (d) V_1/2_ of activation based on data in (c). (e) Voltage dependence of steady-state fast inactivation. Inward current measured during a test-pulse to 0 mV was normalized to the cell’s maximum inward current and plotted against the pre-pulse voltage to fit the data with a Boltzmann function. (f) V_1/2_ of fast inactivation, based on data in (e). (g) Representative trace of the Nav1.7 WT channel peak current amplitude over time when depolarized at 0 mV at 30 Hz. (h-i) Use-dependent current decline at a frequency of 10 Hz (h) or 30 Hz (i). Error bars denote 95% confidence interval. See Table 4 for exact values. These voltage-clamp recordings were performed in the absence of β subunits

We first focused on current density, voltage dependence of activation, and steady-state fast inactivation of the channels (Figure 6), since a change in these parameters could induce a pain phenotype. However, we did not find any change in current density (Figure 6(a, b) (U = 75, p = 0.89), voltage dependence of activation (Figure 6(c, d) (U = 46, p = 0.087), or fast inactivation (Figure 6e, D, see also Table 4) (U = 68, p = 0.61 F). In addition, like for the N1245S variant, no effect was found on either persistent current or time-to-peak of the sodium current (Fig. S3).

**Table 4.** Biophysical gating characteristics of hNav1.7/E1139K. Values are given as mean with upper and lower 95% confidence interval in brackets

	WT	E1139K
Cell number	7–13	9–12
Current density (pA/pF)	461.2 (249.2 to 637.2)	406.2 (279.6 to 532.8)
Activation - V_1/2_ (mV) - Slope	−27.2 (−30.3 to −24.2) 9.8 (9.2 to 10.4)	−30.0 (−32.6 to −27.5) 9.1 (8.7 to 9.6)
Steady-state fast inactivation - V_1/2_ (mV) - Slope	−96.5 (−99.7 to −93.3) -5.6 (−5.9 to −5.3)	−96.5 (−100.0 to −93.0) -6.1 (−6.7 to −5.6)
Steady-state slow inactivation - V_1/2_ (mV) - Slope	−56.6 (−63.3 to −49.9) -11.0 (−12.9 to −9.1)	−59.4 (−68.0 to −50.8) -8.0 (−15.2 to −0.8)
AUC use-dependency at 10 Hz	26.3 (24.9 to 27.7)	26.7 (26.1 to 27.3)
AUC use-dependency at 30 Hz	24.7 (22.7 to 26.7)	24.7 (23.3 to 26.2)

Next, we tested whether the E1139K variant would affect use-dependent current decline in Nav1.7. We used the same protocol as described above, but we limited stimulation frequencies to 10 Hz and 30 Hz. We found that both channel isoforms displayed a similar use-dependent decrease of peak current amplitude, without any difference between the channels at the two frequencies (F(3, 28) = 1.62, p = 0.21) (Figure 6(g-i).

### Nav1.7 kinetics and slow inactivation are unaffected by the E1139K variant

**Figure 7.**
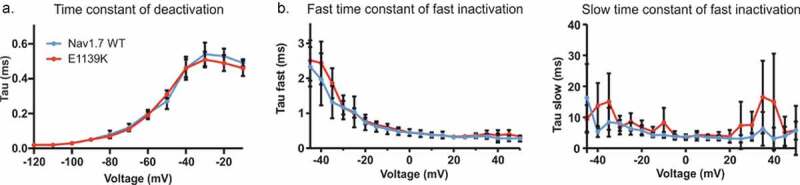
The E1139K variant does not affect the kinetics of Nav1.7. (a) Time constant of deactivation of E1139K (red, n = 10) and Nav1.7 WT (blue, n = 7) channels. (b) Fast (left) and slow (right) component of the time constant of steady-state fast inactivation obtained by double exponential fit. WT in blue, n = 10, E1139K in red, n = 11. Error bars denote 95% confidence interval. These voltage-clamp recordings were performed without co-expression of any β subunit

To investigate a possible effect of the E1139K variant on channel gating kinetics, we used similar recording protocols as described above. Using a single exponential fit to the tail current decay (see also Figure 4c), we found that both WT and E1139K channels deactivated with the same time constant across all voltages, e.g. at −60 mV, E1139K needed 0.19 ± 0. 01 ms and Nav1.7 WT needed 0.20 ± 0. 01 ms to deactivate (Figure 7a). Using a double exponential fit to the current decay during a standard depolarizing pulse (see also Figure 4a), we measured both fast and slow time constants of fast inactivation. Again, both channels displayed the same time constants of fast inactivation (Figure 7b).

**Figure 8.**
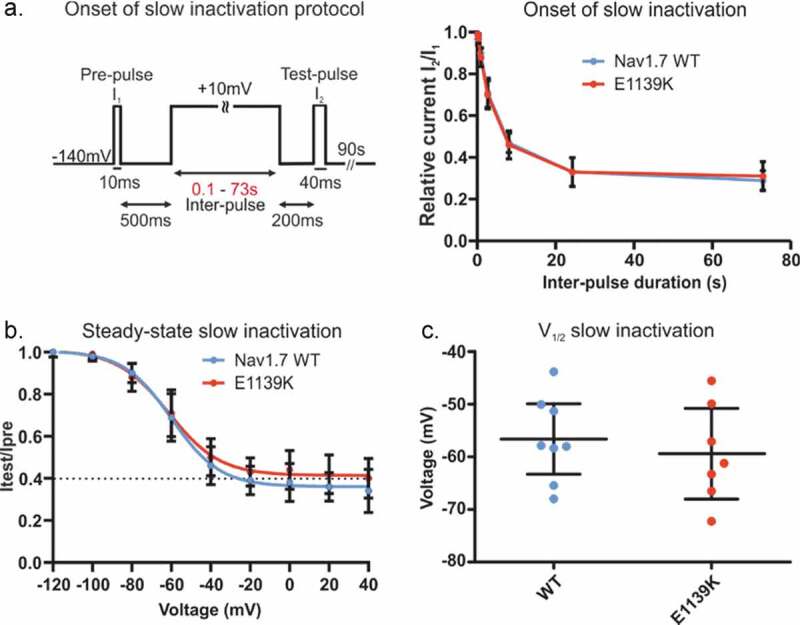
**E1139K and WT channels show similar onset and steady-state slow inactivation**. (a) Voltage protocol of onset of slow inactivation (left) and time course of slow inactivation (right). The peak current at the test-pulse was normalized to the peak current at the ref-pulse and plotted against pre-pulse duration (s) for the E1139K variant (red, n = 11) and the Nav1.7 WT (blue, n = 9) channels. (b) Voltage dependence of steady-state slow inactivation. For protocol, see Figure 5a. Peak current at the test-pulse was normalized to the peak obtained during the pre-pulse and plotted against the pre-pulse voltage to fit the data with a Boltzmann function for E1139K variant (red, n = 10) and Nav1.7 WT (blue, n = 11). (c) V_1/2_ values of slow inactivation were obtained based on data in (b). No difference was observed. Error bars denote 95% confidence interval. These experiments were performed in the absence of β subunits

We have shown above that the N1245S variant enhances slow inactivation. Although it does not directly support a pain phenotype, it is a frequent finding in Nav1.7-IEM mutations [3,23,25] and we were curious to know whether this enhanced slow inactivation may be shared by Nav1.7 variants leading to other persistent pain disorders, such as fibromyalgia. We first measured the time course of the onset of slow inactivation of both the E1139K variant and WT channels (Figure 8). Around 70% of all channels underwent slow inactivation following a 20 s depolarization and the remaining 30% remained available even after a 73 s depolarizing pulse (Figure 8a). Finally, using a 30 s depolarizing pulse to investigate voltage dependence of slow inactivation, we again found no difference between WT and E1139K channels (Figure 8b, c and Table 4).

## Discussion

In this paper, we investigated the potential involvement of two Nav1.7 variants in pain-related disorders: Nav1.7/N1245S and Nav1.7/E1139K. The N1245S variant was found in several patients suffering from IEM or SFN, while the E1139K variant was identified in one patient suffering from severe myalgia exacerbated by exercise. Our voltage-clamp experiments, as well as our 3D computational modeling results for the N1245S variant do not directly support their pathogenicity.

IEM is classically associated with a shift of voltage-dependence of activation of Nav1.7 to more hyperpolarized potentials [26]. In some cases, alterations in other biophysical properties were reported, which is the reason for our in-depth analysis of the presented variants. Fibromyalgia, on the other hand, has so far not been associated with sodium channel mutations yet. The mechanisms underlying fibromyalgia remain poorly understood. A previous study suggested a link between the *SCN9A* single nucleotide polymorphism rs6754031 and the development of fibromyalgia in a group of Mexican women [27]. A study working with a mouse model of fibromyalgia revealed an increased expression level of Nav1.7 in DRGs and the central spinal cord [28]. These two studies suggest a potential involvement of Nav1.7 function in the development of fibromyalgia in patients. However, in our study, we were not able to establish a direct link between the E1139K variant and the pain disorder.

The 3D computer modeling based on the hNav1.7 cryo-EM structure [14] revealed for the N1245S variant one common intramolecular interaction partner and one interaction partner specific to each variant at a distance of 3.2 to 3.5 Å. The hNav1.7 WT interacts with two tryptophan residues, while the N1245S variant interacts with one tryptophan and one tyrosine residue. Thus, the molecular arrangement of the DIII voltage-sensor may be affected by the substitution with the smaller serine. Homology modeling has restraints on the protein backbone and may lead to a falsely underestimated impact on the protein structure by the variant. For a more thorough investigation, molecular dynamics simulations or a structure of the variant would be needed. Based on our 3D homology model, we assume that it is not likely that the variant induces a structural change in the protein conformation of the Nav1.7/N1245S channel and attributes a pathogenic effect to the variant. These data corroborate the voltage-clamp data and tend to confirm the single nucleotide polymorphism identity of the N1245S variant [12].

Although 3D homology modeling is helpful to predict a variant’s potential effect on channel gating, functional assays are needed for a more substantial investigation. Thus, we overexpressed the variants in HEK cells and performed whole-cell patch-clamp experiments. We observed an enhanced slow inactivation in the N1245S variant, which reduces the number of Nav1.7/N1245S variant channels available to open at physiological membrane potentials, thus potentially decreasing neuronal excitability. Many Nav1.7-IEM mutations were described with an enhanced slow inactivation, in addition to other changes in Nav1.7 gating properties [3,22,25,29,30]. Interestingly, IEM mutations with enhanced slow inactivation display impaired resurgent currents in every tested mutation, whereas resurgent currents are always present when probed for in PEPD mutations [25,31]. Thus, the enhanced slow inactivation of the N1245S may indirectly support IEM symptoms, but as no other gating changes were identified in this variant, it alone may not be causative for the disease.

The slow inactivation mechanism is less well understood than that of fast inactivation. Slow inactivation may result from a collapse of the pore region [32]. Although the pore may be the site of conformational changes, the slow inactivation mechanism also requires other regions of the channel, including S5 and S6 of DI and DII, as well as S4 of DIV [18,33,34]. The N1245S variant is located far from these regions (Figure 1), but it may exert allosteric mechanisms involved in the slow inactivation process. Participation of both the voltage-sensing and the pore domains in slow inactivation highlights its intricate development through complex voltage-dependent transitions and suggests that N1245S may impact various regions of Nav1.7. Still, the exact amino acid composition and location of these interactions remain unknown.

Sodium channel β subunits interact with α isoforms at the cell membrane and modulate their membrane expression level and their gating [35]. The β1 subunit is expressed in small and large human DRG neurons [19]. It was shown to form a complex with hNav1.7 in the cryo-EM structure residing close to the DIII voltage sensor [14], suggesting a modulatory function of the excitability of the sensory neurons [19]. According to the same cryo-EM structure, β1 is thought to be close enough to the variant site of hNav1.7/N1245S to potentially interact with it. For these reasons, we investigated the influence of the β1 subunit on Nav1.7/N1245S gating. We found a hyperpolarizing effect of β1 on the activation of WT and the N1245S variant, which may support action potential firing. This similar effect on both channel isoforms suggests that the β1 subunit is not interacting in a specific manner with the N1245S variant.

Several studies highlight an increase of sodium current density in the presence of β subunits [8,35,36]. We also detected a slightly enhanced current density of the N1245S variant compared to the WT channel in the presence of β1 subunit. It is possible that the β1 subunit favors trafficking and thus the expression of the N1245S channel at the membrane level. This may support excitability and pain symptoms. Nevertheless, this effect may be counteracted by the observed enhanced slow inactivation.

β2, β3, and β4 subunits are also found in DRG neurons and modulate the gating properties and kinetics of Nav1.7 [14,19]. The β3 subunit, like β1, is non-covalently associated with Nav1.7 and shares a high sequence homology with β1 [19]. We would expect an increased current density of Nav1.7 WT and N1245S variant channels in complex with β3, as it was previously shown in HEK293 cells [37]. β2 or β4 bind covalently to Nav α subunits via a disulfide bond [35]. A co-expression of either of these β subunits with Nav1.7 did not show any effect on its current density or its gating properties [37,38]. In addition, the binding site for these two β subunits is located too far away from the variant to predict a promising interaction; therefore, we do not expect any change in the gating properties of Nav1.7 in complex with β2 or β4 [39].

Similar to the voltage-clamp results of the N1245S variant, we did not find any change in the gating or the kinetics of E1139K. This variant is located in an intracellular linker, where several mutations leading to IEM, PEPD, or SFN have already been identified [40]. However, sequence alignment with the eight other Nav isoforms showed not only that the E1139 amino acid is not conserved among them, but also that the K1139 is present in the hNav1.3 sequence (Fig. S4). These data suggest that the negatively charged glutamate at position 1139 does not play a key role in the function of Nav1.7 and its replacement by the positively charged lysine does not significantly alter the structure of hNav1.7, neither its function, as shown in our patch-clamp data. The location of this variant within the intracellular DII-DIII linker has not been resolved in the cryo-EM structure, so it remains difficult to predict any possible effect of the variant on overall channel structure or on a possible interaction with β1 or β3 subunits [14]. However, since the E1139K locus is 26 residues distant from the start of DIII S0, where an interaction with β1 seems possible (Figure 1), we assumed that a co-expression of β1 or β3 subunits would not exert any specific effect on Nav1.7/E1139K. To our knowledge, no study has reported a Nav1.7 mutation that is differently regulated by Nav β subunits than the WT channel.

It is possible that the two variants only reveal pathogenic effects when expressed in specific Nav1.7 splice variants. Alternative splicing leads to distinct protein isoforms in Nav1.7 [41]. This mechanism may alter pharmacological sensitivities, kinetics, and channel distribution under pathological conditions [31,42]. This process was shown to occur in Nav1.7 transcripts in human DRG neurons [43]. Human Nav1.7 exists in four different splice variants [44]. Interestingly, these variants showed different levels of expression in a rat model of neuropathic pain, suggesting that the splice variants may play a role in various pain states [41]. Jarecki et al. have shown that Nav1.7/I1461T, leading to paroxysmal extreme pain disorder, shifted activation and deactivation when expressed in different splice variants [31], while another study reported that familial hemiplegic migraine-1 missense mutations attribute different effects on the gating properties of various splice variants of the voltage-gated calcium channel Cav2.1 [45]. Therefore, it may be possible that the two variants tested here could have a pathogenic effect if investigated in a different *SCN9A* transcript variant.

The sense of smell and nociception can influence each other [9,46,47] and seem to have common genes regulating their function, including the *ZFHX2* gene, which encodes a putative transcription factor [48]: a mutation in *ZFHX2* leads to hyperosmia combined with the loss of pain [49]. An even clearer link is provided by the description of patients suffering from chronic insensitivity of pain (CIP). In their case, a complete loss of function of Nav1.7 leads also to anosmia [2]. Thus, the description of hyperosmia in an IEM patient by [9] seemed plausible. However, we did not detect a clear gain-of-function phenotype of the N1245S variant. Other reports could not confirm the link between IEM and hyperosmia [50].

The two rather nonpathogenic variants reported here are not isolated cases in the literature. Many others have been identified, usually classified as having “uncertain significance” or as “not directly pain-causing mutations” [10,51]. Kapetis *et al*. as well as Reimann *et al*., mentioned many *SCN9A* SNPs not related to any pain phenotype in cohorts of patients with painful disorders or osteoarthritis, respectively [12,52]. Cohorts of SFN patients revealed many *SCN9A, SCN10A*, and *SCN11A* variants described as “of uncertain clinical significance” [53]. All these studies, mostly from large cohorts of patients described with painful disorders, attest the existence of many SNPs among Nav1.7, Nav1.8, and Nav1.9 genes not responsible for any pain phenotype, and machine learning-based statistical models point out the difficulty to confirm pathogenicity of channel variants [51].

The whole-cell voltage-clamp technique combined with heterologous expression systems represents a suitable pathway to investigate Nav gating and their pathophysiological role in pain-related disorders. HEK293 cells endogenously express very few ion channels and their Nav TTXs currents are usually small [54]. Nav1.7 is readily expressed and its current amplitude is large enough to assure reliable analysis. However, these cells do also have their limitations and this study shows that such experiments have to be evaluated with care. Cells selected for patch-clamp recordings are usually isolated and do not contain long cellular process in order to avoid space clamp artifacts [54]. HEK293 cells or other heterologous expression systems also clearly do not mimic the native environment of peripheral sensory neurons with all proteins, co-factors, and complex mechanisms associated [55]. It is possible that the N1245S or the E1139K variant expressed in nociceptors undergo post-translational protein modifications, such as glycosylation or phosphorylation, as well as potential Nav dimerization [42,56] that participate in influencing their gating properties in a physiological context. It is thus possible that in a native environment, both variants exert a pathophysiological effect that remains undetected in the biophysical experiments carried out here.

We would therefore like to emphasize that while heterologous expression systems are extremely valuable for biophysical studies of ion channel gating, their results need to be considered carefully and should always be followed by studies in neurons. It is therefore also conceivable that gating changes detected in heterologous expression systems do not necessarily translate to pain pathophysiology in the patient. Accordingly, the opposite could also be true, arguing that the lack of effect seen in this study does not necessarily preclude a pain-causing effect of the two variants in the patients. We summarize commonly used measures to assess a variant's pathogenicity in Table 5.

**Table 5.** Overview of criteria commonly applied to decipher the pathogenicity of a variant. The two Nav1.7 variants investigated here are listed, as well as the pathogenic example of the Nav1.7/I848T erythromelalgia mutation

Nav1.7 variants:	N1245S (this study)	E1139K (this study)	I848T (known pathogenic variant)
cDNA level (NM_002977.3) conservation (nucleotide)	c.3734A>G Highly conserved (phyloP: 7.99 [−20.0;10.0])	c.3415 G > A Moderately conserved (phyloP: 5.75 [−20.0;10.0])	c.2543 T > C Highly conserved (phyloP: 8.94 [−20.0;10.0])
Protein level conservation (amino acid)	p.(Asn1245Ser) Highly conserved, up to Fruitfly (considering 12 species)	p.(Glu1139Lys) Highly conserved, up to Fruitfly (considering 12 species)	p.(Ile848Thr) Highly conserved, up to Zebrafish (considering 12 species)
Allele frequency (carriers/allele), (gnomAD v2.1.1)	1.224/278.244	7/248.222	No frequency
3D modeling shows clash in structure	Slightly	Unknown	Yes [69]
Positive family history	Yes [10]	Unknown	Yes [10]
GOF in voltage-clamp HEK cells	No	No	Yes
Hyperexcitability when expressed in rodent DRG	Not tested	Not tested	Yes [70]
Hyperexcitabilitiy of hiPS cell-derived nociceptor	Not tested	Not tested	Yes [65]
ClinVar entries	RCV000383539.2 (Benign* – Paroxysmal extreme pain disorder), RCV000389147.1 (Likely benign* – Inherited Erythromelalgia), RCV000422016.6 (Benign/Likely benign** – not provided), RCV000335798.2 (Likely benign* – Indifference to pain), RCV000714847.2 (Conflicting interpretations of pathogenicity* – Primary erythromelalgia), RCV000714848.2 (Conflicting interpretations of pathogenicity* – Hereditary sensory and autonomic neuropathy type IIA), RCV000768312.1 (Uncertain significance* – Primary erythromelalgia), RCV000328939.3 (Uncertain significance* – Severe myoclonic epilepsy in infancy), RCV000176065.7 (Benign/Likely benign** – not specified), RCV001080021.1 (Benign* – Hereditary sensory and autonomic neuropathy type IIA).	RCV000553461.2 (Uncertain significance – HSAN type IIA)	RCV000006722.4 (Pathogenic – PE), RCV001004018.1 (Likely pathogenic – Acute episodes of neuropathic symptoms), RCV001067998.1 (Pathogenic – HSAN type IIA)

A potentially more reliable model system would be rodent DRG neurons. Indeed, various studies of Nav1.7 mutants were performed in rodent DRG neurons, where native Nav α subunits are expressed with their native β subunits in a more physiological environment more prone to emphasize their pathogenicity [5,57,58]. However, numerous studies on ion channel gating have shown striking species differences between rodent and human isoforms, leading to a large failure rate in pharmacological development of pain therapeutics [59–64]. Thus, rodent neurons also carry the risk of not representing the physiology of the patient sufficiently well.

To summarize, a more physiological human model may be desirable for the variants to reveal a potential pathogenic function. New possibilities arise from the use of human-induced-pluripotent stem cells (hiPS cells), that retain the genetic background of the donors and can generate any cell type, and their differentiation into sensory neurons [65–68]. This model system allows to study pain-linked variants in their natural environment, i.e. in cells which express all SNPs and variants of the patient’s genome.

Complex prediction based on: https://funnc.shinyapps.io/shinyappweb/

Allele frequency: https://gnomad.broadinstitute.org/

GOF: gain-of-function; PEPD: Paroxysmal extreme pain disorder; IEM: inherited erythromelalgia; HSAN: Hereditary sensory and autonomic neuropathy; PE: Primary erythromelalgia. ClinVar data base entries: https://www.ncbi.nlm.nih.gov/clinvar/.

## Conclusion

Using voltage-clamp and homology modeling experiments, we were able to show that two distinct Nav1.7 variants, identified in chronic pain patients, did not alter the function of the channel to an extent that would directly support the clinically observed pain phenotype. These results suggest that N1245S and E1139K variants are not directly responsible for the pain symptoms of the subjects identified with IEM or fibromyalgia, respectively. Although the whole-cell patch-clamp technique in heterologous expression systems is an adequate method to study genetic variants on their own, it is nevertheless possible that the variants need their native physiological environment to reveal their pathogenic potential. Other proteins may also modulate the variants to show their effect, or the patients may carry another mutation within genes expressed in the DRG neurons or the CNS. These results point to the complexity of Nav1.7, with some mutations leading to clear pain phenotypes, while others have no or only very mild effects on the function of the channel.

## Supplementary Material

Supplemental Material
