# Supplementary material for: Assessing the impact of pain-linked Nav1.7 variants: An example of two variants with no biophysical effect
Source: Channels (Austin). 2021 Jan 25;15(1):208–28. doi: 10.1080/19336950.2020.1870087 (PMC7833769; doi:10.1080/19336950.2020.1870087)
Supplement: Supplemental Material [file KCHL_A_1870087_SM4660.docx]

# Supplementary figures

# Assessing the impact of pain-linked Nav1.7 variants: an example of two variants with no biophysical effect

**Authors**: Kim Le Cann^1^*, Jannis Meents^1^*^#^, Vishal Sudha Bhagavath Eswaran^1^, Maike F. Dohrn^2^, Raya Bott^1^, Andrea Maier^2^, Martin Bialer^3^, Petra Hautvast^1^, Andelain Erickson^1^, Roman Rolke^4^, Markus Rothermel^5^, Jannis Körner^1,6^, Ingo Kurth^7^, Angelika Lampert^1^

^1^Institute of Physiology, RWTH Aachen University Hospital, 52074 Aachen Germany

^2^Department of Neurology, RWTH Aachen University Hospital, 52074 Aachen Germany

^3^Division of Clinical Metabolism of Medical Genetics and Human Genomics at Northwell Health System, New-York, United States

^4^Department for Palliative Care, Medical Faculty, RWTH Aachen University, 52074 Aachen, Germany

^5^Institute für Biology II, Dept. Chemosensation, AG Neuromodulation, 52074, Aachen, Germany

^6^Department of Anaesthesiology, Medical Faculty, RWTH Aachen University, 52074 Aachen, Germany

^7^Institute of Human Genetics, Medical Faculty, RWTH Aachen University Hospital, 52074 Aachen Germany

*Equal contribution

#present address: Multi Channel Systems MCS GmbH, 72770 Reutlingen, Germany

Corresponding authors:

Prof. Dr. Angelika Lampert

Email: [alampert@ukaachen.de](mailto:alampert@ukaachen.de)

Dr. Jannis Meents

Email: [jmeents@multichannelsystems.com](mailto:jmeents@multichannelsystems.com)

Institute of Physiology

RWTH Aachen University

Pauwelsstrasse 30

52074 Aachen

Germany

Tel.: +43 2041 80 88811

Nav1.7/N1245S variant does not show a decreased time to peak or persistent current


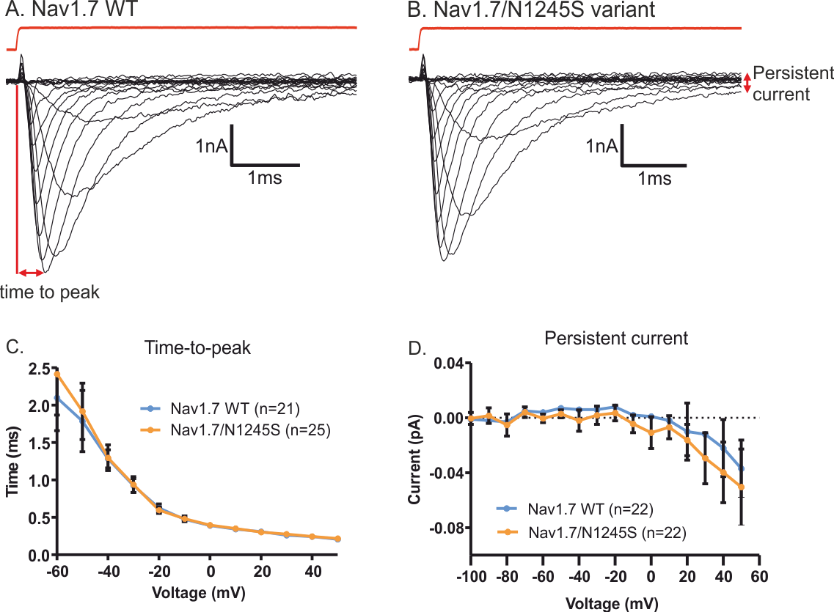


*Figure S1.* ***Nav1.7/N1245S variant has the same time-to-peak than the WT channel and does not show any persistent current****. (A and B) Representative traces of hNav1.7 WT (A) and N1245S variant (B). Voltage stimulus trace depicted in red. Time-to-peak indicated on the WT trace (A) between the beginning of the stimulus and the peak of the sodium current amplitude. (C) Time-to-peak for Nav1.7 WT (blue) and N1245S variant (orange). (D) Persistent current amplitude of Nav1.7 WT (blue) and N1245S variant (orange). Scale 1nA for 1ms. Error bars denote 95% confidence interval.*

Neither N1245S variant nor β1 affects use-dependent current decline


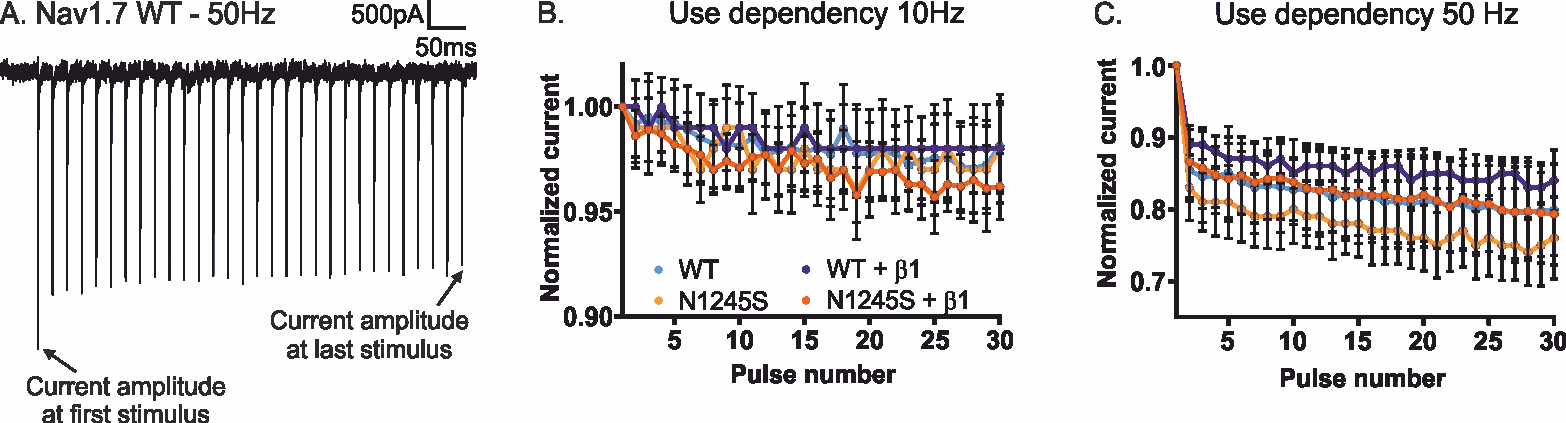


*Figure S2.* ***The use-dependency is similar for the WT and the variant channel N1254S.*** *(A). Representative trace of Nav1.7 WT decline current amplitude at 50Hz. (B-C) HEK293T cells expressing either Nav1.7 WT (n=18), Nav1.7 N1245S (n=17), Nav1.7 WT + β1 (n=18) or Nav1.7 N1245S + β1 (n=16) were stimulated with 30 depolarizing pulses to 0 mV at a frequency of 10 Hz (B) or 50 Hz (C). Inward current measured at each pulse was normalized to the current amplitude from the first pulse.*

Nav1.7/E1139K variant does not affect the time-to-peak or the persistent current amplitude


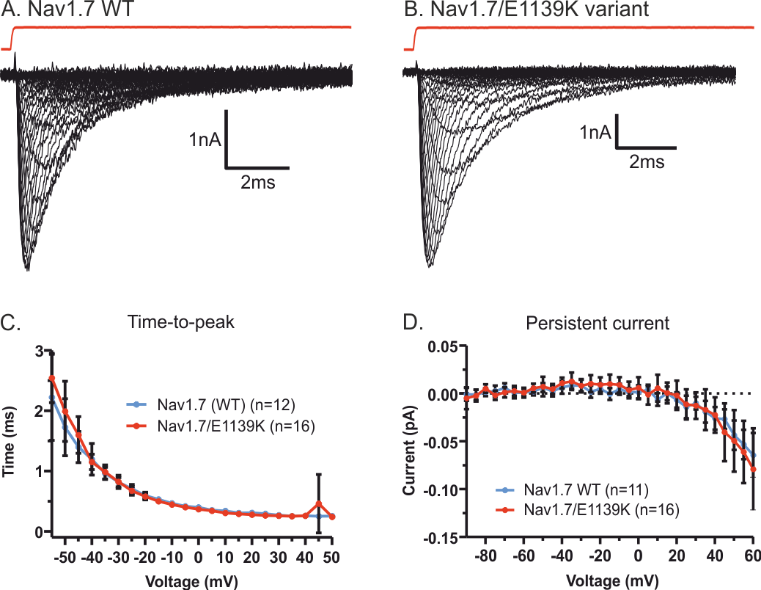


*Figure S3.* ***Nav1.7/E1139K variant reached the maximum peak current amplitude at the same time as the WT channel and does not have persistent current****. (A and B) Representative traces of hNav1.7 WT (A) and E1139K variant (B). Voltage stimulus trace depicted in red. (C) Time-to-peak for Nav1.7 WT (blue) and E1139K variant (red). (D) Persistent current amplitude of Nav1.7 WT (blue) and E1139K variant (red). Scale 1nA for 2ms. Error bars denote 95% confidence interval.*

E1139K is not conserved among Nav protein sequences


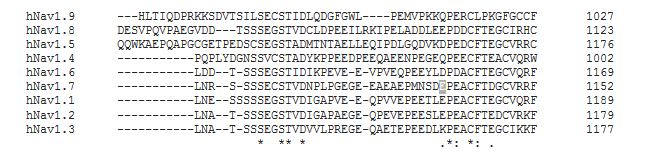


*Figure S4.* ***Nav α subunit alignment sequence reveal that E1139 is not highly conserved among Navs****. Although Nav1.1, Nav1.2 and Nav1.8 contain a glutamate at the position 1139, this amino acid is not found in other Nav sequences. Furthermore, the K1139 of this Nav1.7 pain variant is also found in WT Nav1.3.*

N1245S does not form a novel phosphorylation consensus site

The NCBI reference sequence of *SCN9A* is NP_001352465.1 and the accession number is NM_002977.3 (transcript variant 1 containing 1977 amino acids).

We used the following screening tools:

-NetPhos 3.1 Server: <http://www.cbs.dtu.dk/services/NetPhos/>

-Scansite 4.0: <https://scansite4.mit.edu/4.0/#home>

-Kinase Phos: <http://kinasephos2.mbc.nctu.edu.tw/>

No consensus site of phosphorylation was identified for the variant Nav1.7/N1245S.
